# The Role of Sonic Hedgehog in Craniofacial Patterning, Morphogenesis and Cranial Neural Crest Survival

**DOI:** 10.3390/jdb4030024

**Published:** 2016-08-03

**Authors:** Sebastian Dworkin, Yeliz Boglev, Harley Owens, Stephen J. Goldie

**Affiliations:** 1 Department of Medicine, Monash University Central Clinical School, Prahran, Victoria 3004, Australia; howens@student.unimelb.edu.au (H.O.); stephen.goldie@monash.edu (S.J.G.); 2 Department of Physiology, Anatomy and Microbiology, La Trobe University, Melbourne, Victoria 3086, Australia; 3 Department of Biochemistry and Genetics, La Trobe Institute for Molecular Science, La Trobe University, Melbourne, Victoria 3086, Australia; yeliz.boglev@latrobe.edu.au; 4 Department of Surgery, Monash University Central Clinical School, Prahran, Victoria 3004, Australia

**Keywords:** Hedgehog, neural crest, craniofacial, maxilla, mandible, development, cleft palate

## Abstract

Craniofacial defects (CFD) are a significant healthcare problem worldwide. Understanding both the morphogenetic movements which underpin normal facial development, as well as the molecular factors which regulate these processes, forms the cornerstone of future diagnostic, and ultimately, preventative therapies. The soluble morphogen *Sonic hedgehog* (*Shh*), a vertebrate orthologue of Drosophila *hedgehog*, is a key signalling factor in the regulation of craniofacial skeleton development in vertebrates, operating within numerous tissue types in the craniofacial primordia to spatiotemporally regulate the formation of the face and jaws. This review will provide an overview of normal craniofacial skeleton development, and focus specifically on the known roles of *Shh* in regulating the development and progression of the first pharyngeal arch, which in turn gives rise to both the upper jaw (maxilla) and lower jaw (mandible).

## 1. Introduction—Formation of the Craniofacial Skeleton

The cellular and molecular mechanisms which govern the formation of the vertebrate head and face are remarkably conserved within the entire animal kingdom. Although much of the skull is comprised from mesoderm-derived bone, the majority of anterior craniofacial tissues, including cartilage, bone and musculature are primarily derived from the neural crest cells (NCCs) [1]. NCCs are a population of multipotent, transient migratory cells that are specified at the border of the neural plate and the non-neural ectoderm prior to and during neurulation. In order to form the facial bones, NCCs destined to a craniofacial fate, termed cranial NCCs (CNCCs) migrate ventrally into the frontonasal prominence (FNP), as well as the first, second, third and fourth pharyngeal arches (PA1-4, respectively) [2]. While the forehead and nasal cavities are derived from the FNP, and most facial muscles are formed from PA2, this review will focus exclusively on the first pharyngeal arch, PA1, which primarily gives rise to the maxilla and mandible (upper and lower jaw, respectively), and as such, is a critically important region in the formation of the craniofacial skeleton. Furthermore, this review will focus on the role played by a critical signalling molecule, Sonic Hedgehog (*Shh*), specifically in the context of first pharyngeal arch formation and neural crest survival, development of the maxilla and palatal clefting, and development of the mandible. Other excellent reviews have recently been published which extensively cover the role of *Shh* in the development and aetiology of other craniofacial and neural tube defects [3,4], formation and morphogenesis of the vertebrate teeth [5,6,7], as well as the role of *Shh* partner proteins and pathway components (particularly *Patched*, *Smoothened* and the *Gli* family) in development and disorders of the vertebrate craniofacial complex [8], and as such, these topics will not be covered in this review.

## 2. Development of the Craniofacial Skeleton—The First Pharyngeal Arch

**Figure 1:**
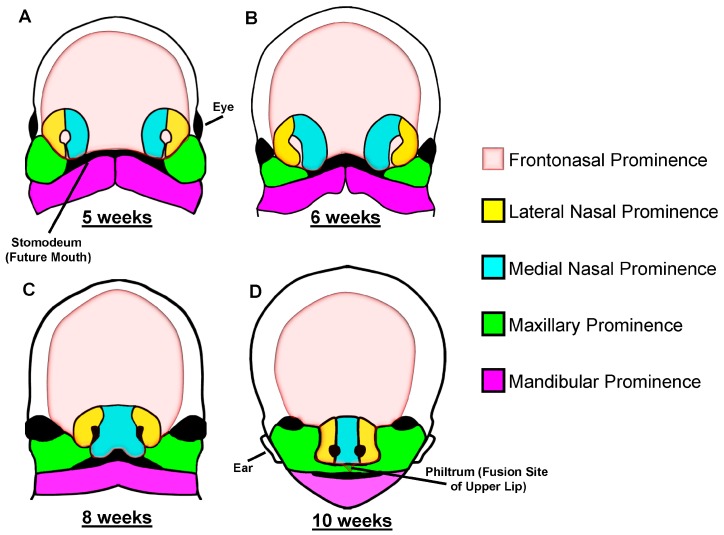
Schematic diagram of human facial development. The craniofacial skeleton is predominantly made up of five primordia; the frontonasal prominence (FNP; including the lateral and medial nasal prominences), paired maxillary (MX), and paired mandibular (MD) prominences. These gradually migrate towards the midline, to form the nose, lips and jaws, and are fully integrated by 10 weeks of human embryonic development. The MD processes appose and fuse first (at ~5 weeks of age; (**A**), followed by fusion of the lateral MX prominences with the medial and lateral nasal processes in the ventrolateral FNP (**B**). Next, fusion of both medial nasal prominences forms the midline of the nose and the primary palate (**C**). Two outgrowths of the MXP (the palatal shelves) elevate and migrate towards each other, fusing with each other and the primary palate to form the upper jaw (**D**). Adapted from [21].

**Figure 2:**
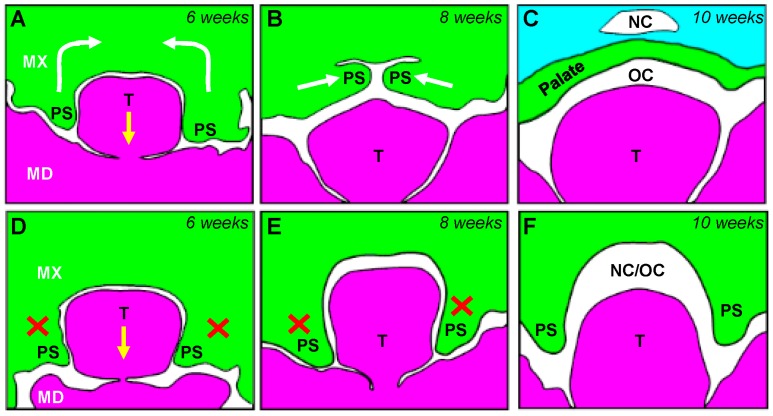
Palatogenesis and development of palatal clefts. The palatal shelves, outgrowths of the maxillary processes, initially form and are directed ventrally, lateral to the developing tongue (**A**). As the embryo develops, the tongue migrates ventrocaudally and the palatal shelves migrate dorsolaterally (arrows in **A**). By 8 weeks of human development, the palatal shelves have begun to migrate towards each other (**B**), and following apposition and fusion, form the secondary palate by 10 weeks, ensuring clear separation between the oral cavity (OC) and, at the anterior end, the nasal cavity (NC; **C**). Although palatal clefting has numerous diverse aetiologies, mechanistically, the palatal shelves remain similarly oriented ventrally at 6 weeks of development (**D**), but fail to elevate (crosses in **D**,**E**). This ultimately results in a failure of secondary palate formation, and a continuous passage between the oral and nasal cavities (at the anterior portion of the face; **F**).

PA1 is the most rostral of the arches, and forms earliest during embryogenesis (approx. embryonic day [E] 8.25 in mouse). In addition to forming the future maxillae and mandible, PA1 also gives rise to numerous other structures, including the malleus and incus bones of the middle ear, the posterior process of the sphenoid bone, the squamous region of the temporal bone, the Masseter muscle, and the mucous membrane and glands of the anterior tongue [9,10]. Having migrated into PA1, CNCCs are induced to proliferate and differentiate by numerous patterning signals emanating from the surrounding pharyngeal environment [11,12,13]. As growth progresses, PA1 becomes segregated into two separate domains, the maxillary and mandibular prominences, which give rise to the cartilage, bones and connective tissue of the upper and lower jaws, respectively [14]. Both prominences consist of bilateral processes that meet on the midline axis and fuse together to form the jaws (Figure 1). For the mandibular processes, this involves epithelial fusion followed by pressure from growth in the apposed mesenchyme pushing out the intervening epithelium, which is incorporated into the oral epithelium that ultimately covers the mandible [15]. Concomitant to further growth of the maxillary processes is regression of the inferior portion of the FNP, resulting in fusion of both medial nasal prominences to form the midline of the nose, the philtrum of the upper lip and the primary palate [16]. During this period, outgrowths of the maxillary process known as the palatal shelves project on either side of the developing tongue. A transient, non-adherent “covering” for the developing oral epithelium, known as the periderm, prevents the opposing epithelial surfaces of the tongue and palatal shelves from inappropriately adhering [17]. Caudal migration of the tongue in response to growth and expansion of the mandible allows the palatal shelves to grow towards the midline and fuse, forming the secondary palate (Figure 1). Fusion of the primary and secondary palates involves growth of the component tissues, epithelial-mesenchymal transition, cell migration and apoptosis at fusion sites [18,19,20]. Perturbation of any of the above processes can potentially lead to failure of primary and/or secondary palatal fusion, and hence either cleft lip with or without cleft palate (CL/P), or isolated cleft palate (Figure 2). Numerous other defects can also occur, as discussed below.

## 3. Defects of Craniofacial Development

All congenital anomalies of the craniofacial structures, regardless of the aetiology or phenotype, are recognized clinically under the broad term of craniofacial defects (CFDs). CFDs are widely prevalent, as they account for approximately 75% of human birth defects, and affect 0.1%–0.3% of all births [1]. The severity of CFDs can range from minor cosmetic irregularities to considerable structural malformations that can lead to problems in breathing and feeding, as well as perturbed social and mental development, and in extreme cases, death. Surgical reconstruction of CFDs is complex, often requiring multiple procedures and not routinely available in many healthcare systems. Therefore, understanding the identity, fate and specification of the cells which comprise the craniofacial skeleton is imperative in order to understand the aetiology of disease. In fact, all CFDs result from either cell-intrinsic defects affecting CNCCs, leading to disorders such as Treacher Collins Syndrome [22] and other neurocristopathies (NCC disorders), or signalling aberrations within the pharyngeal environment, leading to disorders such as Di George Syndrome [23], Fraser Syndrome [24] and Van der Woude Syndrome [25]. Together with a concomitant identification of how CNCCs are instructed to perform their functions by specific (pharyngeal-arch derived) signalling molecules, we can begin to prospectively identify therapeutic windows for intervention. Such avenues for therapy are critical, particularly in much of the developing world, where there is disparity in the incidence of CFDs, compounded with a significant lack of trained surgical, post-operative and supportive care available to repair and treat these debilitating facial anomalies.

## 4. A Key Pharyngeal-Arch Derived Regulator of Craniofacial Development—Sonic Hedgehog (*Shh*)

*Shh* is one of three vertebrate homologues of the Drosophila gene *hedgehog*, first identified in seminal genetic screens by Prof. Christiane Nusslein-Volhard [26], work which culminated in her being awarded the Nobel Prize for Physiology or Medicine in 1995. Along with *Indian Hedgehog* and *Desert Hedgehog*, the vertebrate hedgehog homologues, particularly *Shh*, are among the best-characterised developmental morphogens in vertebrates. First identified in 1993, as a key regulator of polarity, axial development and neural tube floor plate induction [27,28], *Shh* has since been shown to play a multitude of roles in development and disease [29]. Studies conducted in chicken, mouse and zebrafish, as well as analyses of human patients, showed that the *shh* gene is expressed in the notochord, the floorplate of the neural tube, the posterior limb buds and the developing gut [27,28,30,31]. In agreement with its expression pattern, *shh* is important in foregut development [32] and critically involved in patterning of the distal elements of the limbs [28,33,34,35], however, mutations in *Shh* classically lead to defects in midline structures, particularly the face and eyes, and a lack of hemisphere separation of the brain, a disorder termed holoprosencephaly (HPE) [36,37,38,39]. 

The involvement of *Shh* in the aetiology of developmental disorders in human was initially described as the existence of an unknown gene residing within the locus 7q36, a known region implicated in HPE [40]. Patients with HPE also presented with numerous defects of non-neural tube origin, such as ocular hypertelorism, midface hypoplasia, cleft lip only (CLO) and cleft lip/palate [40]. Further studies have shown that a V332A mutation in *Shh* was implicated in a case of solitary median maxillary central incisor syndrome (SMMCI) [41], a novel *Shh* missense mutation (C to T) in the coding region (resulting in a Val for Ala substitution; A43V) was found in a patient with severe median cleft lip/mandible [42], and a 14-month-old girl with submucous cleft palate was found to harbour a duplication of the chromosome region located near the *Shh* gene on 7q36 [43], indicating an important role for *Shh* in human craniofacial development. Outside the facial skeleton, mutations in *Shh* also lead to facial defects of the eye such as coloboma (missing eye tissue) and micropthalmia (abnormally small eyes) [40,44], indicating that *Shh* is a clinically relevant gene in the aetiology of human CFDs. 

## 5. The Role of *Shh* in the Formation of the First Pharyngeal Arch and Maintaining Neural Crest Cell Fidelity

Craniofacial defects caused by *Shh* deficiency largely occur even with correct ventralisation and development of the floor plate of the neural tube (where *Shh* is also expressed) [45], indicating a specific regulatory role for *Shh* within the pharyngeal arches themselves rather than a secondary consequence of neural tube defects. *Shh* is a critical factor for development and survival of cells within PA1, specifically neural crest cells which have colonised the facial prominences [45,46,47]. As a result of CNCC death, mice lacking *Shh* exhibit failure of anterior facial structure formation [48]. Specifically, *Shh*^−/−^ mice demonstrate normal early patterning of PA1 until E9.5, with no concomitant differences in the expression of markers which definitively demarcate the arch endoderm, mesoderm, ectoderm and neural crest cells (*HoxA2*, *HoxA3*, *Dlx3* and *AP2*), as well as markers of pouch identity *Pax1* and *FGF8* [49]. However, within 24 hours, PA1 is greatly reduced in size [49], indicative of global first arch atrophy. As *Shh*-mediated craniofacial defects are largely caused by a loss of neural crest cell fidelity and survival within the arches, [45], these data speak to a critical maintenance, rather than inductive role in the establishment of the correct microenvironment to allow subsequent neural crest cell homing, integration and differentiation. This is supported by evidence showing that in addition to the role of *Shh* in maintaining NCC viability, it also plays an important role in regulating NCC migration [50]. Previous studies implicated *Shh* in the aetiology of Bardet-Biedl syndrome, characterised by facial dysmorphology due to aberrant NCC homing and localisation [51] and using both in vitro cell culture and in vivo models, recent work has shown that mesencephalic NCC migrate to the ocular region as a chemotactic response to an exogenous concentration gradient of the *Shh* morphogen [52]. These data point to a holistic regulation of NCC kinetics by *Shh* within the first pharyngeal arch, subsequent to inductive patterning of the PA1 microenvironment.

**Figure 3:**
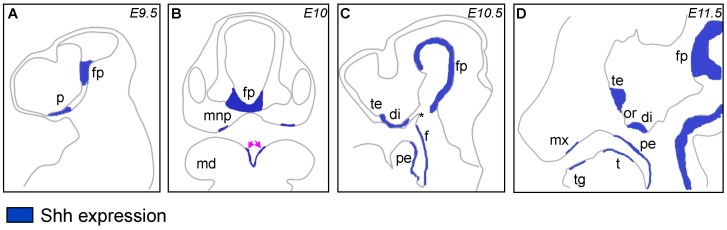
Expression of *Shh* in the developing (mouse) craniofacial skeleton. At E9.5 (**A**), *Shh* is expressed in the ventral-most region of the neural tube, the floor plate (fp) as well as in the ventral prosencephalon (p). At E10, expression within the fp becomes more abundant, and expression is also seen within isolated regions of the medial nasal processes (mnp) as well as the developing mandibular (md) periderm (arrows; **B**). By E10.5 (**C**), *Shh* is localized to the ventral diencephalon (di) and telencephalon (te), as well as in the foregut (f) and pharyngeal endoderm (pe), with notable exclusion from the ectodermal swelling of Rathke’s pouch (asterisk); At E11.5 (**D**), the expression domain of the floor plate extends caudally along the entire length of the neural tube. Expression is maintained within the diencephalon and telencephalon, separated by an exclusion zone at the optic recess (or). Expression is also visible in the epithelium lining the tongue (t), within tooth germs (tg) along the length of both the maxilla and mandible, as well as within the leading edge of the maxillary palatal shelves (mx). Panels **A**, **C** and **D** are sagittal, Panel **B** is coronal. Adapted from [8].

However, *Shh* is expressed widely within the developing PA1, within both the ectodermal and endodermal component, and *Shh* in both these compartments does appear to have some role in later patterning and development of the pharyngeal apparatus, which may be independent in its trophic role supporting CNCCs. Within the ectoderm, *Shh* is expressed within the forming frontonasal prominence and maxillary process from E8.5 in mouse, as well as both primary and secondary palate (Figure 3). Removal of *Shh*-expressing ectoderm (but not adjacent, *Shh*-negative ectoderm) resulted in craniofacial anomalies analogous to human cleft palate [53], indicating that the production of *Shh* within the ectoderm must play a critical signalling function, likely operating concurrently with the production of *Shh* from the endoderm, putatively to establish either paracrine feedback loops or to establish the appropriate concentration gradients to subsequently regulate downstream genetic targets. Within the pharyngeal endoderm of PA1, *Shh* is first expressed at embryonic day (E) 10.5 in the mouse [54], and has also been characterised at an even earlier developmental stage in the chick (HH 9) [55]. This tissue is a critical source of inductive signals for subsequent arch development, as the role of the endoderm in the formation of the pharyngeal arches, and in subsequent patterning of the craniofacial complex has been well-characterised [56,57,58], and numerous studies have shown that a loss of *Shh* within the developing foregut endoderm leads to craniofacial anomalies, characterised by a decreased size and density of the developing facial skeleton [48,59]. These data suggest that *Shh* is important for maintaining overall homeostasis within the developing arch.

In addition to *Shh*, numerous signals and critical genes expressed emanating from the endoderm have been identified, including *fgf3*/*fgf8* [60,61], *vgll2a* [62], *sphingosine-1-phosphate* [63], *Tbx1* [23,64,65], *FRAS1* [24] and *grainyhead-like 3* [66]. It is quite likely that *Shh* interacts with these factors in the context of craniofacial development, either directly, or through co-operative regulation of survival pathways. For example, the common regulation between *Shh* and both BMP4 [67,68,69] and the FGF family [70], particularly FGF8 [54], has been well-described, clearly indicative of paracrine feedback loops. Loss of *sphingosine-1 phosphate* in zebrafish leads to defects in craniofacial skeleton growth, albeit not patterning, defects which are rescued by *shh* mis-expression, and restoration of *fgf8a* [63]. *Tbx1* mRNA transcripts in the pharyngeal endoderm and mesoderm of *Shh*^−/−^ mice were significantly down-regulated, even accounting for differences in arch size [71]. Furthermore, *Shh* appears to act upstream of *Tbx1*, being sufficient to induce *Tbx1* expression following implantation of *Shh*-soaked beads on the surface of the pharyngeal arch in stage 14 chick embryos [71], and *Shh* expression is largely unchanged in E10.5 *Tbx1*^−/−^ mouse embryos [72]. Little is yet known about potential co-operation of *Shh* with *FRAS1, vgll2a* or *grhl3*, however the phenotypes of these morphant/mutant animals (loss of craniofacial structures, primarily due to NCC death or disrupted pharyngeal pouch morphology) suggests that like *shh*, they regulate critical pro-survival pathways subsequent to correct initial arch patterning and specification.

Lastly, several specific mechanisms by which *Shh* mediates CNCC survival, production and apoptosis in PA1 have been described. Primarily, *Shh* regulates palatal mesenchyme proliferation through activation and maintenance of the cell cycle regulators *Cyclin D1* and *Cyclin D2* [70]. Increased expression of the *Shh*-receptor CDO (Cell-adhesion molecule-related/Downregulated by Oncogenes; [73]) similarly leads to neural crest cell death within PA1; elegant in ovo electroporation experiments in chick showed that inhibiting CDO (but not another *Shh* receptor, *Patched*) rescued NCC death in PA1 following loss of *Shh* [74]. High doses of retinoic acid are known to inhibit *Shh* and lead to craniofacial defects [75]. Absence of the apoptosome component *Apaf1* leads to altered *Shh* signalling, mesenchymal hyperproliferation, and delayed skull base ossification [76], indicating that in addition to pro-survival functions, *Shh* may also specifically act to inhibit apoptosis. Despite these advances, and identification of several interacting factors, future work is needed to dissect the entire functional spectrum by which *Shh* maintains NCC survival in PA1.

## 6. Role of *Shh* in Development and Fusion of the Palate (Maxilla/Upper Jaw) and Mandible (Lower Jaw)

By mouse day E11.5, expression of *Shh* is visible within the ectoderm-derived nasal pits, as well as within the lateral-most tips of the future palatal shelves, prior to extension form within the MXP [54]. This expression pattern suggests that *Shh* may be integral to establishing a signalling gradient required for directing subsequent morphogenetic migration of the palatal shelves, and by extension, fusion of the secondary palate. *Shh* continues to be expressed within the palatal shelf epithelium at E13 [77], and is expressed until the two opposing palatal shelves meet and fuse at the midline epithelial seam (MES), although *Shh* is not present during the fusion and subsequent MES breakdown [77]. Interestingly, although the *Shh* receptor *Patched* is expressed throughout the mesenchyme of PA1, *Shh* expression remains largely restricted to the epithelium, indicative of paracrine signalling operating within the arch. As palatal elevation, migration and fusion continues, *Shh* appears to be highly expressed at sites of epithelial thickening within the palate, the segmentation markers, or rugae, suggesting that these are the key sites of *Shh* production in the context of palatogenesis [77,78]. In fact, seminal experiments in avian embryos, in which *Shh*-expressing frontonasal prominence ectoderm was ablated led to maxillary clefts; excision of adjacent, *Shh*-negative ectoderm, did not lead to such defects [79]. These results indicate that *Shh* is a spatiotemporally-regulated signalling molecule, which acts to pattern and specify the palatal shelves.

Unlike the upper jaw which forms from the fusion of separate tissues, the entire lower jaw develops from the mandibular prominences. Clefting of the mandible is rare compared to clefting of the palate and upper lip. More common developmental defects seen in the human mandible are overdevelopment (hyperplasia), underdevelopment (hypoplasia, agenesis and micrognathia) or mis-positioning (retrognathia and prognathism) [76]. Treatment of chick embryos by injection with anti-*Shh* antibody and in mouse embryos injected with jervine, a steroid alkaloid known to inhibit *Shh* signalling significantly disrupts mandibular development [80]. Further mouse models have shown that conditional inactivation of *Shh* in the pharyngeal endoderm leads to reduced lower jaw size (micrognathia) as a secondary consequence of increased neural crest cell death in the first pharyngeal arch [81]. As mentioned previously, much of the role of *Shh* in the first pharyngeal arch is mediated by endodermal *Shh* regulating the expression of FGF8 from within the pharyngeal ectoderm. Loss of either the foregut endoderm [82] or FGF8 within the ectoderm [83] leads to a significant reduction in lower jaw size. Importantly, implantation of *Shh*-coated beads into the pharyngeal region of endoderm-ablated chick embryos restored both FGF8 expression and mandibular development [80], confirming the importance of the *Shh-FGF8* axis in mandibular elongation. 

One of the earliest stages in lower jaw development is the condensation, and differentiation into chondrocytes, of CNCCs to form the hyaline Meckel’s cartilage (MC; [84]). MC development initiates as an aggregation of CNCC-derived mesenchymal cells at the molar tooth bud region, and this structure extends both anteriorly and posteriorly to develop the characteristic “wishbone-like” structure of the pre-mandible. Additionally, the MC also ultimately gives rise to the incus and malleus bones of the middle ear [84]. Whereas these two bones are formed through the process of endochondral ossification (the MC “template” is directly replaced by bone), and the mandible in amphibians and reptiles is also generated by endochondral ossification of the MC [85], the mandibular bone in mammals is formed by a process of intramembranous ossification (whereby CNCC-derived mesenchymal progenitors, surrounding the MC as a fibrous sheath, differentiate into osteoblasts, which subsequently ossify and form the mandibular bones [84,85,86]). In mammals, following osteogenesis, cells of the (anterior) MC primarily undergo degradation before birth [87], however the mesenchymal sheath surrounding the (posterior) differentiated chondrocytes of the MC gives rise to the sphenomandibular ligament [88]. 

The dynamics of MC development and degradation are tightly controlled, as both loss of MC-development or disruption of subsequent MC degeneration (e.g., through inhibition of apoptosis and/or chondrocyte reabsorption) can result in mandibular defects, such as mandibular hypoplasia [84] or a transition to endochondral ossification and subsequent thickening/hyperplasia of the mandible [89]. Therefore, it is clear that any defects affecting CNCC fidelity, both pro- and anti-proliferative, are likely to ultimately impinge on the structural architecture of the lower face, particularly with respect to subsequent patterning and morphogenesis of the mandible. 

Following removal of *Shh* responsiveness specifically in the mouse neural crest cells, Jeong and colleagues discovered that the primary requirement for *Shh* signalling was in post-migratory craniofacial development, rather than the initial generation and migration of neural crest cells [90]. Given that *Shh* signalling is essential for ectomesenchymal cell proliferation and survival and that Meckel’s cartilage development is dependent on CNCC-derived cells, these data indicate that the absence of Meckel’s cartilage in *Shh* null mice is due to an insufficient number of neural crest-derived cells within the mandibular arch, a defect and mechanism highly consistent with the aforementioned roles of *Shh* in maintaining neural crest cell fidelity in PA1 in the context of palatal development. Thus, following loss of *Shh*, increased apoptosis results in apparent aplasia of Meckel’s cartilage, an early pathogenic event.

Taken together, these studies indicate that *Shh* is an important regulator of both maxillary and mandibular development, primarily through its role in maintaining a sufficiently adequate critical mass of CNCC-derived ectomesenchymal cells within the entirety of PA1 to allow for subsequent correct morphogenesis and patterning of the craniofacial skeleton.

## 7. Conclusions and Future Directions

The strong co-operative and functional interactions between *Shh* and both retinoic acid and ethanol indicate that embryos with abrogated or hypomorphic *Shh* signalling may ultimately benefit from modulation of the retinoic acid pathway, and also be highly susceptible to ethanol exposure, as well as potentially other environmental insults. Interestingly, craniofacial defects induced by ethanol share remarkable similarity with those caused by *Shh*-deficiency, causing significant death of cranial neural crest cells [91] and decreased *Shh* transcription [92]. Ethanol also severely impacts on the ability of *Shh* to act as a chemotactic agent in vivo, inhibiting the migration of cranial neural crest cells [52]. It is therefore tempting to speculate that modulation of the *Shh* pathway may ameliorate the severity of some of these defects [92], providing an extremely promising new avenue for the potential limitation of not only *in utero* craniofacial defects, but also for decreasing the severity of Fetal Alcohol Spectrum disorders. As alluded to earlier, determining the molecular pathways (primarily cell survival/apoptosis) active in *Shh*-mediated regulation of CNCC fidelity will prove critical to therapeutic interventions. 

Numerous environmental factors are associated with the aetiology of CFD, including exposure to ionising radiation, vitamin deficiency, tobacco smoke and maternal alcohol intake [93]. It is likely that embryos with a genetic predisposition to CFD will suffer much more severe defects from exposure to environmental factors than embryos without any sensitising mutations. Characterising such gene-environment interactions (GEI) is crucial, as genetic mutations could then be accurately used as a pre-natal diagnostic tool to limit the severity of peri-natal birth defects. Limited GEI data is available; predominantly derived from large-scale analyses of human CFD populations [93], and very few animal models of CFD susceptibility to environmental insult currently exist. The identification of genes such as *Shh*, which both regulate craniofacial development, and are themselves influenced by environmental factors, will therefore significantly shape future therapies of severe pre-natal defects of human development.
